# Adapting a Motivational Interviewing Intervention to Improve HIV Prevention Among Young, Black, Sexual Minority Men in Alabama: Protocol for the Development of the Kings Digital Health Intervention

**DOI:** 10.2196/36655

**Published:** 2022-07-13

**Authors:** Henna Budhwani, B Matthew Kiszla, Angulique Y Outlaw, Robert A Oster, Michael J Mugavero, Mallory O Johnson, Lisa B Hightow-Weidman, Sylvie Naar, Janet M Turan

**Affiliations:** 1 Department of Health Policy and Organization School of Public Health University of Alabama at Birmingham Birmingham, AL United States; 2 School of Medicine University of Alabama at Birmingham Birmingham, AL United States; 3 Wayne State University Detroit, MI United States; 4 Department of Medicine University of California San Francisco San Francisco, CA United States; 5 University of North Carolina at Chapel Hill Chapel Hill, NC United States; 6 College of Medicine Florida State University Tallahassee, FL United States

**Keywords:** HIV, men who have sex with men, pre-exposure prophylaxis, youth, implementation science, intervention, Alabama, African American, sociocultural, structural barriers

## Abstract

**Background:**

African American or Black young men who have sex with men (BYMSM) are at a disproportionate risk for contracting HIV and have high rates of undiagnosed, and therefore untreated, HIV infection. In the southern United States, BYMSM face region-specific hurdles to HIV prevention, such as limited access to care and high levels of racism and intersectional stigma, necessitating HIV testing and pre-exposure prophylaxis interventions that address sociocultural and structural barriers while motivating BYMSM to engage in prevention. Brothers Saving Brothers (BSB) is a motivational interviewing behavioral intervention that successfully and simultaneously increased community-based HIV testing and prevention counseling and education among BYMSM in the midwestern United States.

**Objective:**

The aim of this protocol is to detail the process for the adaption of the BSB intervention for midwestern BYMSM to the Kings intervention for southern BYMSM. During the adaptation process, the intervention will be modernized to include rapid HIV testing, as opposed to HIV testing that requires BYMSM to return for test results, pre-exposure prophylaxis, and the provision of structural supports, and for relevance in the southern United States.

**Methods:**

Aim 1 is to gather qualitative data through focus groups and in-depth interviews with BYMSM aged 18 to 29 years in Alabama and in-depth interviews with prevention and outreach workers who routinely work with BYMSM in Alabama. NVivo qualitative software (QSR International) will be used for the coding and analysis of the transcripts via a thematic analysis approach. For aim 2, intervention mapping will guide the adaptation process, intervention content, components, and design. Both aims 1 and 2 will leverage the Exploration, Preparation, Implementation, Sustainment implementation science framework, with emphasis on the exploration and preparation phases of this model. By applying these frameworks, the original midwestern BSB intervention will be scientifically adapted to the southern BYMSM Kings intervention.

**Results:**

This study is ongoing as of 2022 and is expected to conclude in 2024, with aims 1 and 2 being completed in 2023. Qualitative data will offer insight into the current real-world experiences and preferences of BYMSM in Alabama. Feedback will be collected through the adaptation process to inform intervention refinement. Institutional review board approvals have been received.

**Conclusions:**

The findings will inform next steps, that is, testing the Kings intervention for feasibility, acceptability, and preliminary effectiveness in a pilot hybrid type 1 effective-implementation randomized controlled trial. The study results will provide insights about important considerations for HIV prevention among BYMSM in the southern United States.

**Trial Registration:**

ClinicalTrials.gov NCT03680729; https://clinicaltrials.gov/ct2/show/NCT03680729

**International Registered Report Identifier (IRRID):**

PRR1-10.2196/36655

## Introduction

### Background

Over the past decade, African American or Black young men who have sex with men (BYMSM) have seen a doubling of new HIV infections [1-3]. The Centers for Disease Control and Prevention and state public health departments concur that 70% to 75% of new youth infections are linked to condomless anal sex, and these infections are concentrated among BYMSM (54.4%) [1-5]. The Centers for Disease Control and Prevention also report that BYMSM have the highest rate of undiagnosed, and therefore untreated, HIV infection [3,4,6]. Young men who have sex with men (MSM) are less likely to know their HIV status compared to older MSM [7]. The proportion of MSM who are unaware of their HIV status is highest among African American and Black populations (59%) and lowest among those who identify as White (26%) [7,8]. Nationally, over half of MSM have not been tested in the past year; these rates are lower in the southern United States [1,3,5,9], making studies that address barriers to testing and prevention among BYMSM critical and urgently needed to address the epidemic, particularly in the southern United States.

BYMSM in the Deep South—a subgroup of southern states—face significant barriers to HIV testing and prevention. The seven Deep South states—Texas, Georgia, Alabama, South Carolina, Mississippi, Florida, and Louisiana—have populations that hold more conservative values; are more rural, with less access to health care; and tend to embrace strong religiosity that stigmatizes sexually active youth and MSM [10]. The Deep South holds some of the highest rates of HIV and poverty in the country, and these rates are pronounced among southern racial minorities [1,3,5,7-14]. Southern BYMSM are at greater HIV risk than peers living elsewhere in the United States due to sociocultural factors, including stigma related to HIV and same-sex behaviors, structural racism, and limited health care infrastructure [7-21]. Stigma and structural racism are consistently associated with HIV risk behaviors as well as negative health outcomes [7-11,22,23]. Interventions that combat sociocultural and structural barriers to HIV prevention have the potential to motivate BYMSM to routinely test and consider pre-exposure prophylaxis (PrEP).

Considering the urgent need to address the HIV epidemic among BYMSM in the southern United States, in this protocol, we detail the adaptation of the Brothers Saving Brothers (BSB) HIV prevention intervention for midwestern BYMSM to the Kings intervention for southern BYMSM.

### The BSB Intervention

BSB is a 2-part, face-to-face counseling intervention that was developed and tested in Detroit, Michigan, that aims to improve rates of community-based HIV testing, the return for HIV test results, and prevention education among BYMSM. BSB was developed by using Information-Motivation-Behavioral Skills (IMB) theory, and it delivers messaging with developmentally tailored motivational interviewing (MI). In the first part of BSB, using MI, BYMSM are offered an orientation to HIV (“HIV 101”), are encouraged to accept HIV testing in the community, and return for test results. The second part of BSB is conducted after the participant has tested, if the participant returns for test results, and if the test result was nonreactive. In the second BSB module, the outreach worker shares the test results and offers extended HIV prevention education with MI. Each part is 20 to 30 minutes long (total: 40-60 minutes). In the Michigan BSB trial (trial number: H97HA0378; N=188), participants in the intervention group were more likely to engage in community-based HIV testing (49% vs 20%) and return for HIV test results (98%) compared to the control (72%; *χ^2^*_1_=10.22; N=65; *P*=.001) [24,25].

### Kings Intervention

In the adaptation of BSB to Kings, the following changes will be made. First, while the 2-part format will be retained and the first module will continue to be focused on promoting HIV testing, in Kings, the second module will promote PrEP uptake instead of being focused on prevention education, which was the focus in the original BSB intervention. Second, BSB was delivered in person within community settings. Kings will be delivered digitally in community settings. Third, BSB was tailored to leverage the language of the urban Midwest; Kings will employ language that is commonly used in the southern United States. Both BSB and Kings use IMB theory and tailored MI. More information on the adaptation process is provided in the *Methods* section.

### Summary of Scientific Premise

To our knowledge, BSB is one of the few behavioral interventions that successfully and simultaneously targets community-based HIV testing and prevention education among BYMSM, warranting adaptation for BYMSM in the southern United States. If BSB is successfully adapted to Kings, the Kings intervention could improve rates of testing and prevention services, thereby preventing HIV acquisition in the southern United States. Thus, the purpose of this protocol is to detail the steps within the following two research aims: (1) elucidate experiences, beliefs, and perspectives related to the delivery and utilization of HIV testing and prevention services for BYMSM and (2) adapt the BSB intervention to include two HIV prevention tools (rapid testing and PrEP).

## Methods

### Ethics Approval

This protocol, including all associated data collection tools and informed consent forms, was reviewed and approved by the University of Alabama at Birmingham Institutional Review Board (approval number: IRB-300002136).

### Theories and Tools for Behavior Change

#### Behavior Change Theory

This study is guided by the IMB model [26-31]. BSB, when adapted to Kings, will aim to improve knowledge of HIV prevention and PrEP (information) and self-efficacy (motivation), resulting in HIV testing and PrEP initiation when appropriate (behavioral skills to enact behavior change). Enhanced knowledge will provide BYMSM with the information they need to make evidence-informed decisions about their own health. Enhanced motivation is protective against stigma, which is routinely experienced by BYMSM, and enhanced behavior skills will enable BYMSM to access HIV testing, PrEP, and health care that is imperative to their well-being. IMB will inform the development of the standardized in-depth interview and focus group guides for aim 1, and the intervention components will map to IMB domains for aim 2.

#### Implementation Science Framework

The Exploration, Preparation, Implementation, Sustainment (EPIS) implementation science framework [32-34] will be used to inform this protocol. EPIS [32-34] can be used to move behavioral interventions from development or adaptation to full-scale and ongoing practice by building understanding of relevance and applicability to new contexts and through identifying factors that affect dissemination, adoption, integration, sustained use, and the impact on target populations. During the early phases of EPIS, which are relevant to this study, the model stresses intervention users’—BYMSM and HIV prevention and outreach staff—beliefs and impressions of the intervention and the inner and outer contexts affecting routine practice (aim 1). Therefore, during the dissemination of the intervention (aim 2), we can take these factors into consideration to increase the likelihood of acceptability, feasibility, and effectiveness, which result in longer-term sustainability [7,8,32-36].

#### Overview of MI

MI is a highly specified behavior change communication approach to improving relationships between clients and providers [37-40]. Miller and Rollnick [41] state the following:

MI is a collaborative, goal-oriented style of communication with particular attention to the language of change. It is designed to strengthen personal motivation for and commitment to a specific goal by eliciting and exploring the person’s own reasons for change within an atmosphere of acceptance and compassion.

MI promotes behavior change and treatment engagement across a range of behaviors [42-44]. MI’s emphasis on autonomy support and its ability to address apathy toward behavior change makes it an optimal evidence-based approach to embed within behavioral interventions for youth [45]. MI is already included in clinical guidelines for HIV care and HIV risk reduction in the United States [46]. Given the benefits of MI in BSB, MI will be retained in the Kings adaptation.

### Protocol Inclusion Criteria and Recruitment

The study participants will be BYMSM aged 18 to 29 years and HIV prevention and outreach workers aged ≥18 years who are of any race, gender, sex, and orientation. All participants must reside in the state of Alabama.

We are collaborating with two community-based organizations. Birmingham AIDS Outreach provides services to people in the Birmingham metropolitan area and is affiliated with the Magic City Wellness Center [47]. Selma AIDS Information and Referral provides services to populations in rural Alabama and refers clients to the Medical Advocacy and Outreach clinics in Selma, Montgomery, Dothan, and Atmore, Alabama [48]. These agencies will recruit eligible participants from their catchment areas through health fairs, clinic-based recruitment, and flyers.

### Needs Assessment

The needs assessment phase was guided by aim 1—elucidate experiences, beliefs, and predictors related to the delivery and utilization of HIV testing and prevention services for BYMSM by using qualitative research methods to inform the adaptation of BSB (ie, *Exploration* in EPIS).

#### Qualitative Guides

Informed by the EPIS framework, the IMB model, and prior research on HIV prevention in the southern United States and HIV prevention with young sexual and gender minorities, the team developed standardized guides—a focus group guide for BYMSM, an in-depth interview guide for BYMSM, and an in-depth interview guide for HIV prevention and outreach workers. The guides cover a standardized set of domains, specifically HIV prevention barriers and facilitators, stigma, culture, racism, structural factors, and COVID-19. The guides were developed by using language at an eighth-grade reading level, and prompts focused on elucidating the sociocultural environment that may affect how the intervention is accepted and received [32,34,49-54]. The guides were pilot-tested with key informants for acceptability related to language, content, and length. The prevention and outreach worker interview guide is provided in Multimedia Appendix 1.

#### Data Collection With BYMSM

Focus groups and interviews were conducted with BYMSM (estimated sample size: n=36-48). BYMSM selected their preferred modalities—focus groups or in-depth interviews that were conducted either face-to-face via the web-based, Health Insurance Portability and Accountability Act–compliant Zoom platform (Zoom Video Communications Inc) or by phone call. Face-to-face in-depth interviews and focus groups were conducted at our community-based organization partners’ sites in a private room to protect confidentiality. The target sample sizes are provided, but data collection continued until data saturation. An African American or Black research assistant conducted in-depth interviews and focus groups. An example question set was “What do we need to do to get rid of barriers to HIV testing? What would help?”

#### Data Collection With Prevention and Outreach Staff

Face-to-face in-depth interviews were conducted with HIV prevention and outreach staff (estimated sample size: n=10-12) from both community-based organizations. Topics specific to these interviews included views on how youth-friendly and minority-friendly their services are, approaches to discussing PrEP and prevention with clients, opinions on how to increase the utilization of prevention services by African American or Black youth and BYMSM, and the sociostructural support services that are available or are needed. The principal investigator conducted these interviews on-site at community-based organizations in a private room. Example questions included “How do you explain the importance of HIV testing to clients” and “What kinds of issues do you encounter with promoting HIV testing?”

#### Analytical Methods

Qualitative in-depth interviews and focus groups were audio-recorded by using digital recorders, and audio files were uploaded to an encrypted server. Audio files were transcribed into Microsoft Word by Landmark Associates. Coding and analysis involved applying a thematic analysis approach [55], in which a priori themes and subthemes from theory and literature are supplemented with emerging themes “grounded” in data [56]. Two experienced coders cocoded transcripts by using NVivo software (QSR International). As a first step, a preliminary coding scheme was developed based on the literature and the topics included in the interview guide, such as HIV prevention barriers and facilitators, stigma, culture, racism, structural factors, and COVID-19. This coding scheme was used and appended during the initial review and coding of the transcripts, resulting in a refined coding scheme. Transcripts were then rereviewed and were made more detailed; second-level fine coding was conducted. As of June 2022, analytic reports have been compiled; analyses are underway.

### Intervention Design

The intervention design phase is informed by aim 2—adapt the BSB intervention to include HIV rapid testing and PrEP, address structural barriers, and be acceptable to BYMSM in Alabama (ie, *Preparation* in EPIS).

#### Intervention Mapping

**Figure 1:**
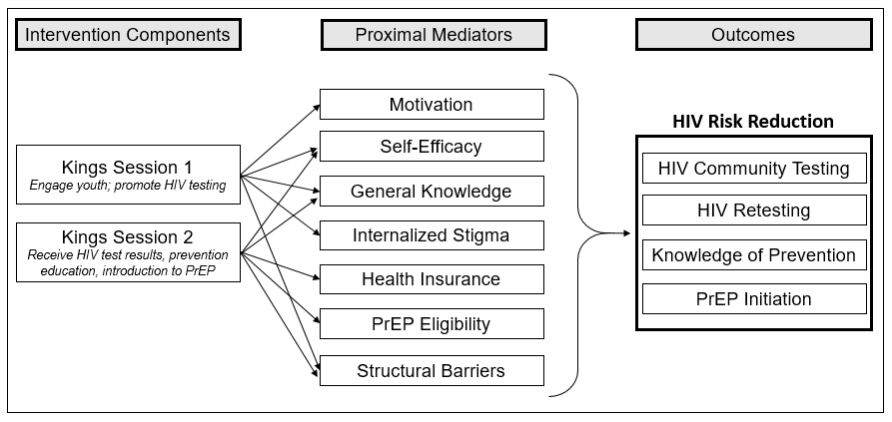
Intervention mapping framework for adapting the Brothers Saving Brothers intervention to the Kings intervention. PrEP: pre-exposure prophylaxis.

The 4-step intervention mapping model [57] will be employed to guide the adaptation process. Using intervention mapping will enable us to prioritize key targets while considering barriers to testing and prevention. The steps are needs assessment, change objectives, intervention design, and production (Figure 1), which are described as follows:

1. Needs assessment: This step is part of aim 1—qualitative data collection from both stakeholders and beneficiaries.
2. Create change objectives: Change objectives relate to the behavior change targeted by the intervention. BSB has 2 parts, necessitating 2 objectives. The first is to increase HIV testing, and the second is to increase PrEP uptake.
3. Intervention design: This step includes decision-making about the intervention's structure based on the knowledge generated for aim 1. Since a premise of intervention mapping is that all intervention components must be informed by theory, it should be noted that we will continue to use the IMB framework that informed the creation of the original BSB intervention for this proposed modernization and adaptation.
4. Production: This includes the adaptation of intervention components and intervention pretesting.

#### Digital Delivery

There is a strong body of evidence that suggests that the digital delivery of HIV-related interventions is preferred by young MSM and sexual and gender minorities [58], especially in high-stigma or remote settings. Additionally, our data for aim 1 indicated that the most acceptable way to deliver Kings is via the internet; thus, Kings will be developed as a 2-session digital health intervention that is built by using web-based MI [59].

## Results

The tasks outlined in this protocol will be completed in 2023. Aim 1—qualitative data collection—concluded in 2021. As of June 2022, the aim 2 adaptation process is underway.

## Discussion

### Study Overview

We anticipate that this study will provide two outcomes. The first is insight (provided via the conduct of aim 1) into how to motivate and support BYMSM in accepting HIV testing and engaging in HIV prevention in the unique environment of the southern United States. The second is the development of a testable HIV prevention digital health intervention.

### Limitations

Although our study is poised to make a positive impact on the HIV prevention continuum among BYMSM in the southern United States, African American or Black transgender women are not included in this project. Transgender women are also disproportionately affected by HIV in Alabama and therefore may benefit from an HIV prevention intervention that is tailored to their lived experiences. Of note, this protocol is being conducted in part during the COVID-19 pandemic, which will likely affect outcomes and influence how the BSB intervention is adapted for current contexts and sociopolitical circumstances. Although our team selected intervention mapping to guide the adaptation process, there are other frameworks that could have been applied; these frameworks could have potentially resulted in different intervention modifications.

### Future Directions

The immediate future directions are to complete the analysis of the aim 1 qualitative data, finalize the adaptation of the digital health intervention, and evaluate the adapted Kings digital health intervention with BYMSM in Alabama.

## Appendix

Multimedia Appendix 1 Interview guide for HIV prevention and outreach staff (example).

## Abbreviations

BSB: Brothers Saving Brothers
BYMSM: Black young men who have sex with men
EPIS: Exploration, Preparation, Implementation, Sustainment
IMB: Information-Motivation-Behavioral Skills
MI: motivational interviewing
MSM: men who have sex with men
PrEP: pre-exposure prophylaxis

## Data Availability

The data that will be generated during the study will be available from the corresponding author on request.
