# Supplementary material for: Adapting a Motivational Interviewing Intervention to Improve HIV Prevention Among Young, Black, Sexual Minority Men in Alabama: Protocol for the Development of the Kings Digital Health Intervention
Source: JMIR Res Protoc. 2022 Jul 13;11(7):e36655. doi: 10.2196/36655 (PMC9330190; doi:10.2196/36655)
Supplement: Multimedia Appendix 1 [file resprot_v11i7e36655_app1.docx]

| **Qualitative Interview Guide for Prevention and Outreach Staff** | |
| --- | --- |
| **Theme** | **Interview Questions** |
| **Introduction (Rapport)** | 1. Can you tell me a little about your organization and your role in your organization? 2. What are some of the best things about your organization? |
| **Transition** | Now that we have gotten to know each other a little better, I would like to move into some of the main topics that we want to cover in this interview. |
| **Approach to HIV Testing** | **Overall Current Status of HIV Testing**   1. Tell me about your organization’s approach to HIV testing 2. How do you explain the importance of HIV testing to clients? 3. What kinds of challenges do you encounter with HIV testing? 4. Why do you think some people decline HIV testing? What the common reasons people provide when declining an HIV test?   **Community Based Testing**   1. How about HIV testing in the community? Please describe the process. 2. Where do you currently conduct HIV testing? 3. Can you please describe any training you have received to conduct community based HIV testing? 4. Tell me about the characteristics of the team that conducts community-based HIV testing. 5. How do you think the rates of HIV testing by BYMSM could be improved?   **Structural Barriers to HIV Testing**   1. In your opinion, what are some barriers to getting HIV tested by BYMSM? 2. What do you believe can be done to address these barriers? 3. How do you think the MSM community perceives these barriers? |
| **Transition** | Thank you so much. I appreciate you sharing your thoughts. Next I would like to spend some time talking about your organization’s approach to engaging youth, sexual and gender minorities, and people of color. |
| **Engaging People of Color and Youth** | **Youth**   1. Tell me about your organization’s approach to engaging youth, those under 29? How would you break down the age categories? What are the issues for each age sub-group? 2. How does your organization reach out to youth? 3. Tell me about your experiences working with youth in your role at your organization. 4. How do you think young people feel about HIV? What do they know about HIV and risk reduction? 5. What are some routinely asked questions by youth about HIV testing and PrEP?   **Young People of Color**   1. Tell me about your organization’s approach to engaging Black youth. 2. How does your organization actively reach out to racial minorities? 3. Tell me about your experiences working with racial minorities while at your organization.   **Sexual and Gender Minorities**   1. How does your organization actively reach out to sexual and gender minorities? 2. How do you feel the Black MSM community feels about your organization? |
| **Transition** | I mentioned PrEP in the prior set of questions, because as someone working at a non-profit, I know you are aware of PrEP. In this next section, we will talk a bit about PrEP and barriers to both HIV testing and PrEP. |
| **PrEP** | **General**   1. What are your thoughts on PrEP? 2. What does your organization do to promote PrEP? 3. Do you receive any support for your PrEP programs? If so, please tell me about how they contribute to your PrEP programs. 4. How do you explain PrEP to clients? 5. Why do you think more Black MSM are not on PrEP in Alabama?   **Linkage to Care**   1. Please describe your process to link potential clients (e.g. for confirmatory testing, for PrEP) to care outside of your own organization.    1. How long does it take you to get an appointment for a client at your preferred partner clinic?    2. Describe the typical issues encountered when linking clients or potential clients to care.   **Structural (Community Supports)**   1. Tell me about any community-based supports to access testing and PrEP? 2. Are there programs to help poorer people with basic needs in the community? 3. What kinds of supports does your agency offer? 4. Does the community know about these supports? |
| **Transition** | This has been great. In this third and last section, we will discuss culture and things I should consider in adapting the Brothers Saving Brothers (BSB) intervention.  *Demonstrate “chunking” and “ask-tell-ask” from BSB.*  Do you have any questions before we start? |
| **Culture and Adapting BSB for this Cultural Context** | 1. How would you describe southern culture? 2. How would you describe gay culture in the South? 3. Help me understand how Black MSM experience living in the South? 4. What role, if any, does religion play in shaping the culture and experiences of sexual and gender minorities in the South? 5. What should an HIV testing and PrEP intervention consider when trying to promote HIV testing and PrEP to BYMSM in the South without being inappropriate? 6. What type of outreach workers do you think can best connect with BYMSM? |
| **Conclusion** | Is there anything I have not asked you about that you think I should know about? (i.e., what have I missed in this conversation?) Thank you very much for your time! Your responses will be very helpful for improving HIV testing and PrEP services for BYMSM in Alabama and hopefully, eventually, across the Deep South. |
